# Undersaline endoscopic resection via antral submucosal tunnelling for a postpyloric duodenal lesion

**DOI:** 10.1055/a-2944-3694

**Published:** 2026-09-03

**Authors:** Alessandro Gubbiotti, Gemma Maddalo, Lorenzo Guido Wilde, Erik Rosa Rizzotto

**Affiliations:** 1 Gastroenterology and Endoscopy Unit Padua 18625 Ospedale Sant'AntonioPaduaVenetoItaly; 2 Gastroenterology and Endoscopy Unit 18703 Ospedale Santa Maria di Ca FoncelloTrevisoVenetoItaly; 3 9308 University of PaduaPaduaVenetoItaly

Superficial non-ampullary duodenal epithelial lesions (SNADELs) are being reported more frequently 1​ and thought to carry a non-negligible risk of progression to adenocarcinoma. 2​ Hence, a proper endoscopic resection, although challenging in this setting, is mandatory to prevent invasive surgery.

Endoscopic mucosal resection (EMR), despite safer, leads to lower en bloc resection and R0 rate compared to endoscopic submucosal dissection (ESD); in contrast, duodenal ESD has been found to carry worse outcomes, as opposed to colorectum and upper digestive locations. 3​ ​4​ Different ESD strategies have been suggested in SNADELs to overcome technical challenges, such as the pocket-creation method, traction method, and water pressure method with saline immersion. 4​ Recently, a new ESD approach via submucosal tunnelling (ERAST) has been described, to deal with SNADELs with unfavorable anatomical approach, such as peri-pylorus location. 5​

**Fig. 1:**
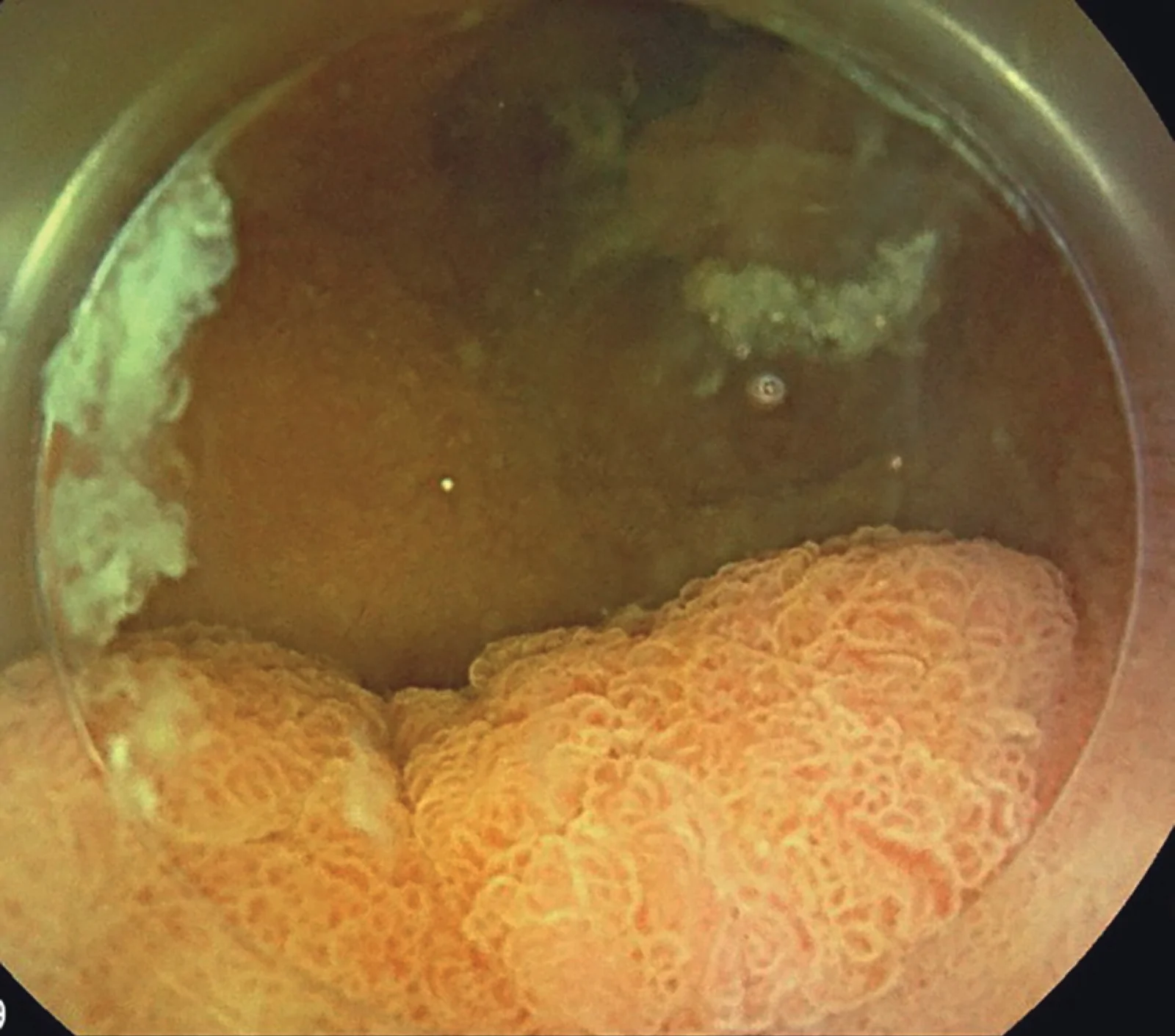
Undersaline vision of the lesion, sited just beyond pylorus. Undersaline enhances vision assessment owing to its floating effect, even in unfavorable locations.

We present a case of a 63-year-old male patient, with a previous surgical history for colorectal-cancer and distal pancreas metastasis, referred for the incidental diagnosis of a 20 mm postpyloric elevated lesion, along a posteroinferior wall ( Fig. 1 ), resulted in high-grade dysplasia (HGD) on forceps sampling.

**Fig. 2:**
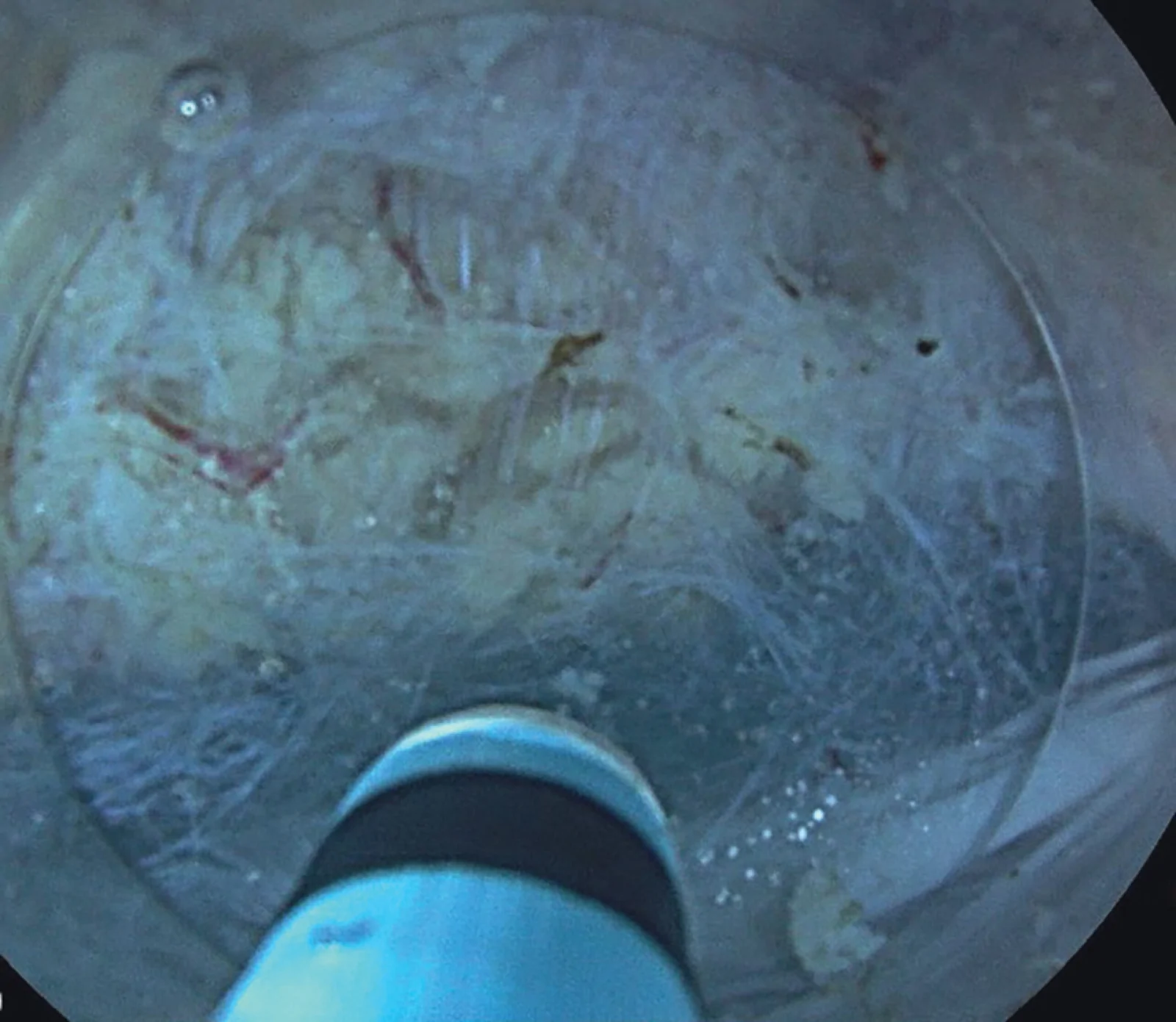
Undersaline submucosal dissection through the duodenal bulb. The submucosal layer, although narrow, is clearly differentiated from the muscle and mucosal layer.

**Fig. 3:**
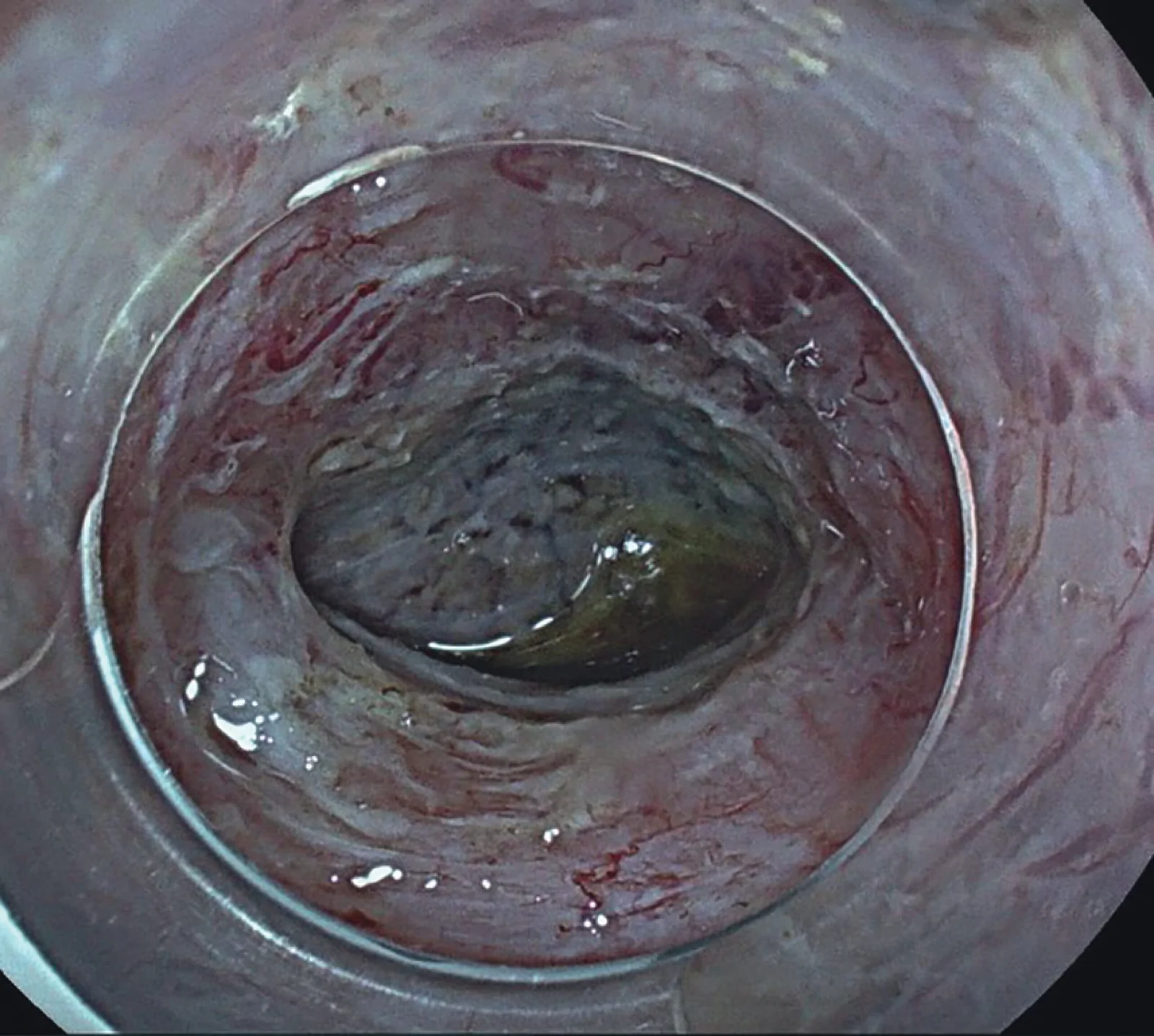
Final picture of the submucossal tunnel and pylorus muscle, showing no defects nor residual perforating vessels.

**Fig. 4:**
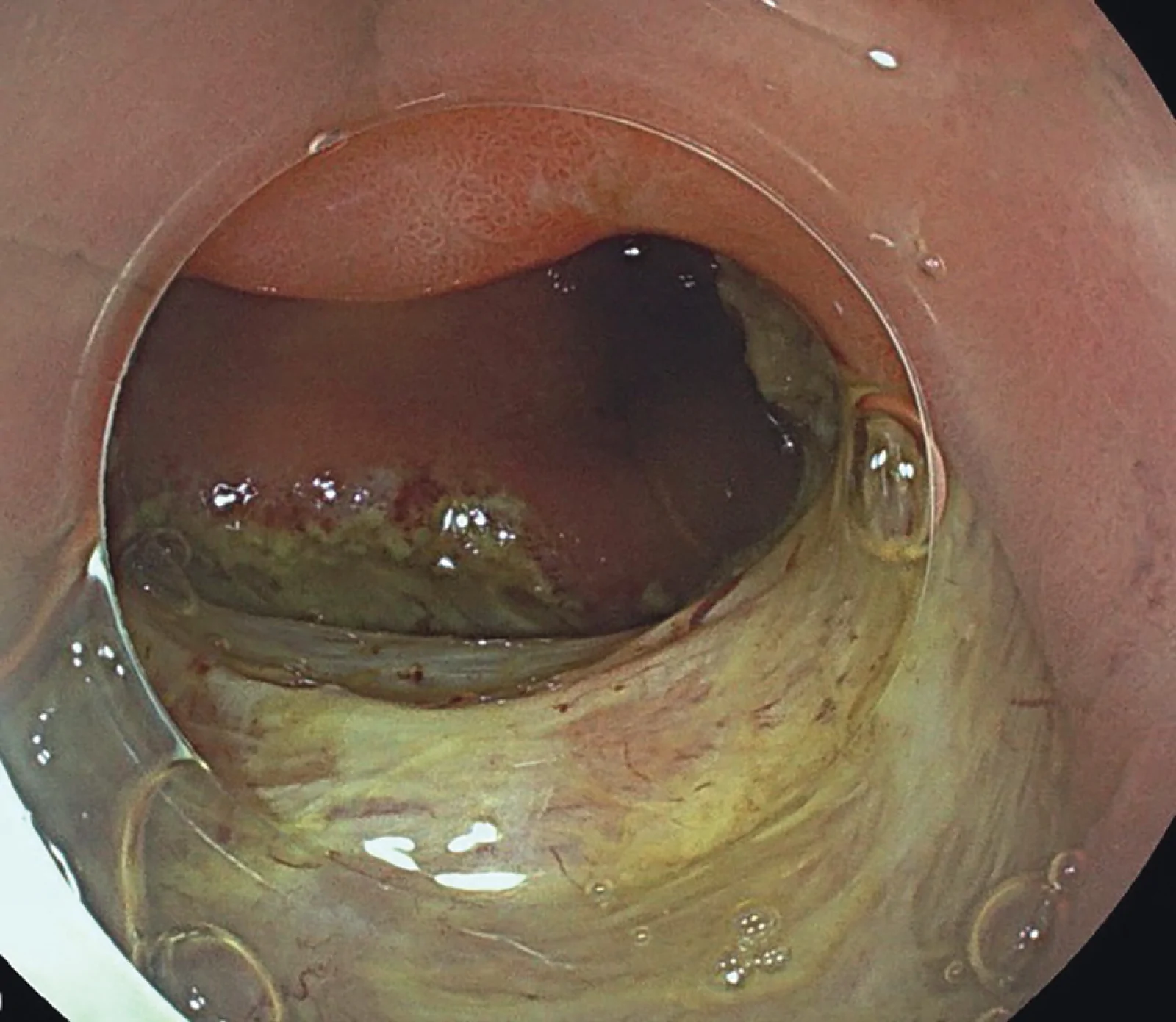
Final mucosal defect encompassing the bulb, pylorus and antrum.

**Fig. 5:**
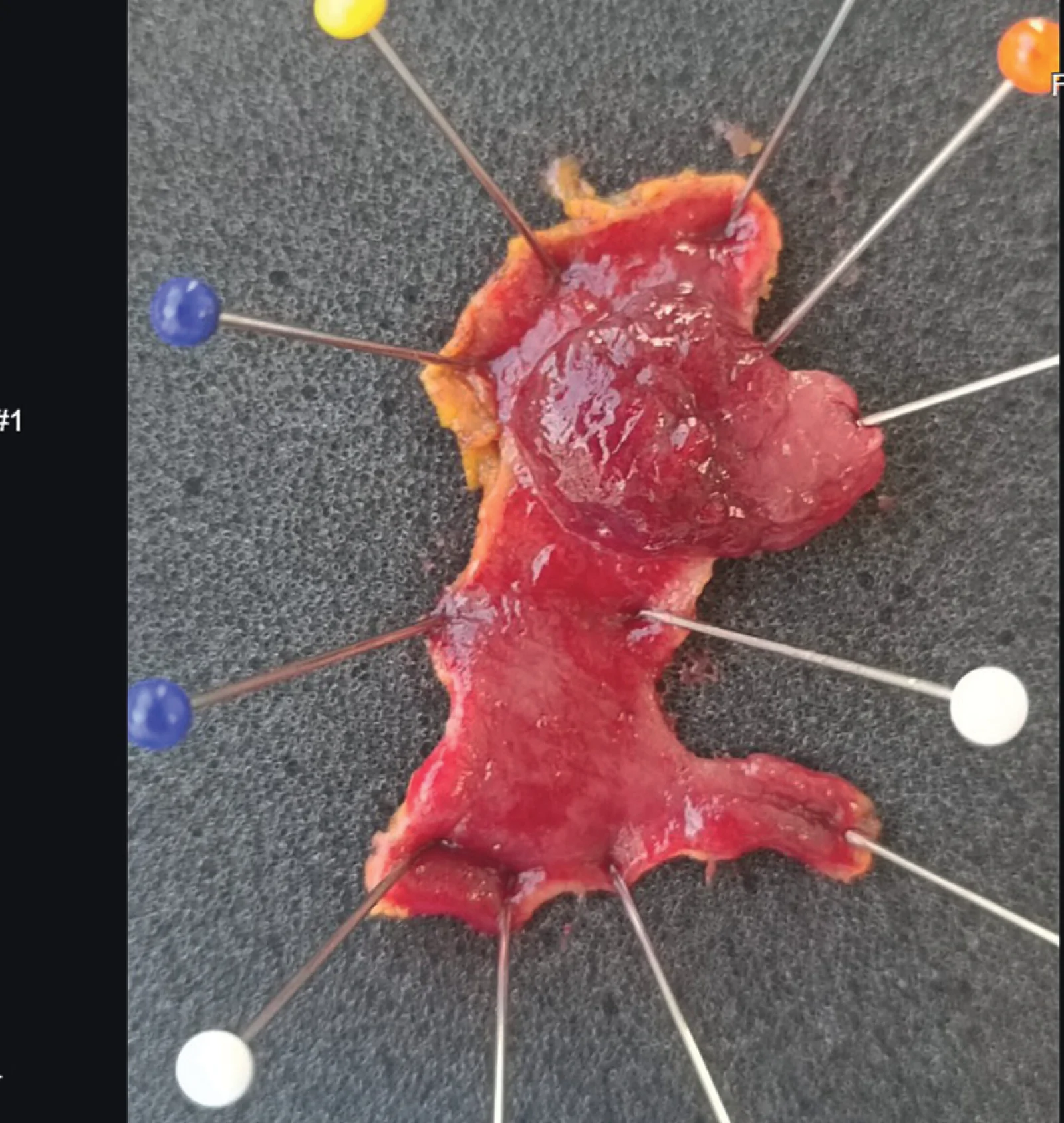
Specimen orientation necessary for a proper histological assessment of lateral margins.

The location prevented the EMR approach with the curative purpose so that we proceeded with a combined undersaline-ERAST strategy (U-ERAST), under general anesthesia ( Video 1 ). First, we performed an antral submucosal tunnelling until pylorus, therefore switching to undersaline dissection ( Fig. 2 ), to achieve better submucosal exposition, scope handling and vessel management ( Fig. 3 ), through the duodenal bulb. Undersaline also facilitated anal incision, to complete the tunnel ( Fig. 4 ), and, eventually, lateral margin incision, with no need for further traction. The patient was discharged on day 2, with uneventful clinical course. HGD with clear margins (R0) was confirmed on the histology specimen ( Fig. 5 ).

**Video 1** Undersaline endoscopic resection via antral submucosal tunnelling (U-ERAST) for a postpyloric duodenal lesion. Video URI: .


Our combined approach achieved safe and curative resection, preventing further invasive treatments in a patient with surgical history. Combining strategies might be a key to overcome anatomical hardships in SNADEL resection, even with ESD, in selected cases.
